# A Comparison between the Tie-over and Closed Suction Drainage Therapeutic Strategies in Patients Suffering from Sacral Pilonidal Sinus

**Published:** 2016-12

**Authors:** Mojtaba Ahmadinejad, Koorosh Ahmadi, Izadmehr Ahmadinejad, Amir Masoud Hashemian, Peyman Khademhoseini

**Affiliations:** 1 General Surgery Department, Alborz University of Medical Sciences, Karaj, Iran; 2 Department of Emergency Medicine, Alborz University of Medical Sciences, Karaj, Iran; 3 Iran Atomic Energy High School, Iran; 4 Department of Emergency Medicine, Mashhad University of Medical Sciences, Mashhad, Iran

**Keywords:** Sacral Pilonidal Sinus, Tiover, Closed Suction Drainage, Cyst, Surgery

## Abstract

**Background and Objectives:**

Pilonidal sinus is a disease in the sacrococcygeal region diagnosed through the purulent discharges of the above-said region. Although the exact pathology and etiology of those suffering from pilonidal is not clear yet, the presence of hair seemingly plays a major role in the process of infection and the granulation tissue. Several techniques have been identified for pilonidal surgery. These techniques primarily fall within two categories: Primary repair and Lay open. One of the setbacks of the primary repair method is the creation of a dead area under the wound which can result in blood accumulation and seroma. To solve the problem of removing the dead space, there are two solutions. The first method utilizes a close suction drain, when the wound discharges are over, the drain is removed. The second technique is called Tie-over where different layers of the wound are pushed close to one another and the dead region vanishes. The present research seeks to compare Tie-over and Closed Suction Drainage methods through random clinical trial in order to introduce the superior technique for faster recovery and reduction of the economic load on the patient.

**Methods:**

Some 64 patients suffering from sacral plonidal sinus aging from 15 to 50 in Shohadaye Ashayer Hospital of Khoram Abad in 2013 were selected for the research based on the inclusion criteria. The demographic information of them was collected through questionnaires. The patients were randomly divided into two groups undergoing (A) Tiover and (B) Closed Suction Drainage surgeries. Other information such as return to normal activity and total recovery time was also completed through the questionnaire. SPSS software was used to conduct statistical analysis.

**Results:**

The results of the statistical analysis showed that the two groups were similar to one another in terms of age, gender, marital status, job, and literacy. A significant difference was observed only between the level of satisfaction (*P*-value = 0.035) in groups A and B concerning the factors studied after the operation. The post-operation recurrence of pilonidal sinus among those who smoked cigarettes and had undergone Closed Suction Drainage was significantly greater than the non-smokers (*P*-value=0.011). As of the group undergoing Tie-over surgery, the difference between the patients’ satisfaction in terms of their age was statistically significant and the highest level of satisfaction was observed among those aging 25 to 34 (90%) (*P*-value=0.023).

**Conclusion:**

In sacrococcygeal pilonidal cyst surgery, no difference was observed except for the difference in the level of satisfaction. More satisfaction was observed using the Tiover method.

## INTRODUCTION

Pilonidal sinus is a disease in the sacrococcygeal region diagnosed through the purulent discharges of the above-said region. These discharges leak from one or more holes in the center line of the above-said region. These holes are connected to a sinus located between the skin and sacral fascia. These holes are sometimes shut off and the discharges accumulate in the sinus. This will result in an abscess formation in the region. The only treatment for pilonidal sinus in the chronic or acute state is surgery, but there is still no consensus about selecting the preferred surgery method to treat pilonidal sinus (1-4).

Out of every 100 thousand people, 26 are afflicted with pilonidal sinus. It is 4 times as prevalent among men as women. The peak age for catching this disease is 16 to 20 years old and it is less observed after 40 (5).

Although the exact pathology and etiology of pilonidal disease is not clear, the presence of hair seemingly plays a major role in the process of infection and the granulation tissue (6).

It is assumed that while sitting, suction is caused in the center line which results in the inward movement of the hair. This hair grows inward and might result in infection and present itself as an acute abscess in the sacrococcygeal region. After the acute stage is removed, recurrence of the wound is quite common (6).

This disease is mostly observed among hairy people and those with sitting jobs are usually reported to be suffering from it. It is thus called the drivers’ disease (7). 

The greatest causes of pilonidal sinus are infection, injuries, and hair being trapped in the deep tissues of the coccyx region. This problem is mostly experienced during the puberty age when the growth of hair and activity of sebaceous glands grows high (7).

Pilonidal sinus mostly affects the youth of a society and it is one of the major causes that disrupt their usual life. Those afflicted with this issue have difficulty undertaking even the simplest activities in their life. Due to its high rate of recurrence and the long time required to treat the surgical site, this disease is one of the most important causes that prevent the youth to go to work (8). Concerning the pathogenesis of this disease, two theories which consider it to be congenital or acquired have been proposed where the acquired theory has gained greater acceptance (9).

Various techniques have been proposed to for pilonidal surgery which mostly falls into 2 categories: Lay open and primary repair. One of the setbacks of the primary repair method is the creation of a dead area under the wound which can result in blood accumulation and seroma (10). To solve the problem of removing the dead space, there are two solutions. The first method utilizes a close suction drain, when the wound discharges are over, the drain is removed (11).

The second technique is called Tie-over where different layers of the wound are pushed close to one another and the dead region vanishes. In the closed repair method, the wound is simply stitched after the excision or the wound is repaired and healed using plastic surgery techniques such as using flaps or Z-plasty. As of those patients who suffer from the recurrence of the disease, the wound has to be totally removed and left open so that it can be healed using Secondary Healing, or flaps need to be used to fill the wound. Using flaps is rarely necessary (12).

The difference between return to work among those pilonidal sinus patients operated through Tie-Over and Closed Suction Drainage is the main motif of this research. The present research seeks to compare Tie-over and Closed Suction Drainage methods through random clinical trial in order to introduce the superior technique for faster recovery and reduction of the economic load on the patient due to absence from work and recurrence of the disease.

## MATERIALS AND METHODS

Some 64 patients suffering from sacral plonidal sinus in Shohadaye Ashayer Hospital of Khoram Abad in 2013 were selected for the research. All the male and female patients aging 15 to 50 who were diagnosed with sacral pilonidal sinus who had filled the consent form and were not afflicted with diabetes, immune system weakness, cancer, and collagen vascular disease entered the study.

To collect the information, a questionnaire containing the demographic particulars of the patients and questions concerning the any background diseases, smoking cigarette and history of disease was utilized. The patients were randomly and equally divided to two groups, namely A (surgery using Tie-Over techniques) and B (surgery Closed Suction Drainage).

The patients in both groups A and B were hospitalized in the hospital and received Cefazolin 1 gram half an hour before operation as prophylaxis. Then, all the patients underwent spinal anesthesia and were operated in the prone position.

As of the patients in group A who had undergone Tie-over surgery, first an Elliptical incision was afflicted on their skin and the sinus was fully removed up to presacral fascia. Then, using a coater, the subcutaneous tissue and skin were removed from the Medius and two flaps were created on both sides of the wound. After the hole was fully rinsed and the blood was removed using the coater and when there was no bleeding, the skin and subcutaneous and the whole presacral fascia were stitched using 3 Tension stitches using 0-1 cut nylon in the upper, middle and lower sections. After this stage, the released flaps were sewed to one another using absorbable suture (0-2 Vicryl), then subcutaneous tissue were pushed close to one another. In the final stage, skin layers were pushed close to one another using 0-3 nylon with far and near stitch so that the edges of the wound got Evert. Then, a bandage was placed on the wound and fastened using Tention stitches.

Close Suction Drainage technique was used for group B. The same steps taken to remove the injury for group A were repeated here and pilonidal sinus was completely removed. After the hole was completely rinsed, No. 14 hemovac drain was placed inside and got out of the upper-outer section of the incision and a coater was used to suck the blood from the wound. Next, 0-2 Vicryl was used to push the subcutaneous close to each other and the skin was repaired using 0-3 nylon. After the operation, the patients were taken to the hospital ward and stayed there for 24 hours receiving Cefazolin (injected) and 25 mg PRN (Pro re nata) Pethidine.

The patients were visited by the surgeon in the first week after operation and checked for side effects of surgery such as infection and bleeding. Then, Tension stitch and Hemovac drain were removed. The skin stitches were opened on the 14^th^ day in both groups and the follow up continued for 6 months and the remaining information including return to normal activity and total recovery was collected using a questionnaire.

Then, the data was fed to SPSS software and descriptive statistical methods (mean and standard deviation, ratios and percentages) and independent T analytical statistics or its parametric equivalent and Chi-square tests were used to analyze the information.

## RESULTS

This research studied 64 patients suffering from pilonidal sinus who were operated in Shohadaye Ashayer Hospital of Khoram Abad in the first half of 2013 using Closed Suction Drainage and Tie-over techniques. The average age of the patients operated using the Closed Suction Drainage was 28.2 ± 9.2 years, while this average for those who had undergone Tie-over surgery technique was 29.5 ± 10.8 years old. The results of statistical analysis demonstrated no significant difference between the two groups in terms of age, gender, marital status, employment, and the literacy level. This proves the consistency of the groups studied. The following factors were studied among both groups after the operation: occurrence of pain (*P*-value=0.18), frequency of taking pain killers (*P*-value=0.56), wound infection (*P*-value=0.45), occurrence of bleeding (*P*-value=0.52), recurrence of pilonidal sinus (*P*-value=0.55), recurrence of injury (*P*-value=0.2), level of satisfaction (*P*-value=0.035), and total recovery and absence from work period (*P*-value=0.091). A significant difference was observed only between the level of satisfaction in groups A and B. The results are presented in Table 1.

**Table 1:** A comparison between post-surgery satisfaction levels in the groups studied

post-surgery satisfaction	Low Number (%)	Average Number (%)	Much Number (%)	Total Number (%)	*P*-value

The groups studied					

Closed Suction Drainage	5 (15.6)	19 (59.4)	8 (25)	32 (100)	0.035
Tie-over	2 (6.3)	12 (37.5)	18 (56.3)	32 (100)	

The difference in occurrence of wound, bleeding, recurrence of pilonidal sinus, and the post-surgery level of satisfaction based on the sitting style in both groups was insignificant. It was also discovered that the difference in the above-said factors after surgery in both groups in terms of the style of lying down was also insignificant. These results and their insignificance was also repeated based on the patients’ gender. No significant difference was in terms of acne in both groups (*P*-value=0.39).

No statistically significant difference was observed in wound infection, bleeding and level of satisfaction after operation in terms of smoking cigarette, but the post-operation recurrence of pilonidal sinus among those who smoked cigarette and had undergone Closed Suction Drainage was significantly greater than non-smokers (*P*-value=0.011) (Table 2).

**Table 2:** A comparison between post-surgery satisfaction and side effect levels in the groups studied based on smoking cigarette

Operation	Smoking cigarette	Wound infection		Bleeding		Recurrence		Satisfaction		
		yes	no	yes	no	yes	no	low	average	high

Closed Suction Drainage	yes	2 (22.2)	7 (77.8)	2 (22.2)	7 (77.8)	1 (1.1)	8 (98.9)	0	5 (55.6)	4 (44.4)
	no	3 (13.6)	9 (86.4)	5 (22.2)	17 (77.8)	1 (4.5)	21 (95.5)	5 (22.7)	14 (63.6)	3 (13.6)
	*P*-value	0.76		0.97		0.011		0.1		
Tie-Over	yes	1 (12.5)	7 (87.5)	1 (12.5)	7 (87.5)	1 (12.5)	7 (87.5)	0	4 (50)	4 (50)
	no	4 (16.7)	20 (83.3)	6 (25)	1 (75)	1 (4.2)	23 (95.8)	2 (8.3)	8 (33.3)	14 (58.3)
	*P*-value	0.77		0.64		0.39		0.55		

After separating the patients into three age groups (15-24, 24-34, older than 35), it turned out that the difference in the level of satisfaction in the group undergoing Tie-over surgery was statistically significant in terms of the patient’s age and the highest level of satisfaction was observed among the age group 25-34 (90%) (*P*-value=0.023).

## DISCUSSION

Pilonidal sinus is a multi-factorial disease mostly observed among men. Some researches concerning the techniques for operating pilonidal sinus have been published but none has been introduced as the golden standard. The essays published over the last 4 decades point to the full excision of pilonidal sinus as the favorable therapeutic strategy (13). There are also many surgeons who emphasize on excision and primary stitching as the best methods to treat acute pilonidal sinus (14). The present research seeks to determine a simple and less dangerous method which yielded the best results among the patients resorting to Shohadaye Ashayer Hospital of Khoram Abad.

The average age of the patients in our survey was 28.85, while in the study conducted by Mc Callum *et al*., an average age of 21 was reported for guys and an average age of 19 was reported for the ladies suffering from pilonidal sinus (15). In another study conducted by Ghafouri *et al*., the average age of the patients was 25.47 ± 7.9 years old (16).

75% of the patients in our research were male and 25% were female. In the study conducted by Ghafouri *et al*., the majority of the patients were male (87.5%) (16). In the study conducted by Sane’i *et al*., 92.7% of the patients were male (17). The majority of the patients were male in the study conducted by Mc Callum *et al* (15). These results confirm the greater prevalence of this disease among young men.

The difference between the recurrence frequencies in the Tie-over and Close Suction Drainage techniques were 1.3 and 3.6 percent respectively. The insignificance of this correlation has also been confirmed by Sane’i *et al*. The results of their study indicated that the relative frequencies in the Tie-over and Close Suction Drainage techniques were 7.3 and 5.5 percent respectively (17). In another study conducted by Holembac *et al*., a recurrence was observed among 17% of the patients operated using Tie-over technique (18).

As our results indicate, the difference in the cases of wound infection, bleeding and post-surgery satisfaction based on smoking cigarette was not significant among those who had undergone Closed Suction Drainage surgery, but higher levels of pilonidal sinus recurrence was observed among smokers than what was observed among non-smokers. Surgical site infection was observed among 15.6% of those undergoing Close Suction Drainage and among 9.4% undergoing Tie-over and this difference was not statistically significant. According to the studied conducted by Holembac *et al*., patients undergoing Tie-over surgery experienced surgical site infection in 43% of the cases (19).

Concerning the frequency of taking pain killers, the patients in the Tie-over group (71.9%) and those in the Close Suction Drainage group (78.1%) required painkillers less than 3 times indicating the little pain experienced in both techniques. In the study conducted by Ghafouri *et al*., the average post-operation pain in the Close Suction Drainage was significantly correlated with more satisfaction and less pain on the side of the patients (16).

The results indicate a high rate of satisfaction among those undergoing Tie-over surgery, while merely 25% of those undergoing Closed Suction Surgery had high levels of satisfaction. This indicates a statistically significant difference in post-operation levels of satisfaction in both groups. The difference in levels of satisfaction in terms of their age was statistically significant with the highest level of satisfaction observed among the age group 25-34 (90%). However in the study conducted by Toccaceli *et al*, the primary closure of the pilonidal sinus using Close Suction Drainage was reported to yield good results in terms of recovery level, morbidity, quick return to work, recurrence level and patients’ satisfaction. It is thus introduced as the favorable method for treating plonidal sinus (20).

## CONCLUSIONS

In a nutshell, the results of our study show no difference in the final results of operating sacrococcygeal pilonidal cyst using the tie-over and Closed Suction Drainage, except for levels of satisfaction. As the results of our study indicate, the post-operation satisfaction levels associated with the Tie-over technique were significantly higher than Closed Suction Drainage.

## References

[1] Al-Khamis A, McCallum I, King PM, Bruce J (2010). Healing by primary versus secondary intention after surgical treatment for pilonidal sinus. Cochrane Database Syst Rev.

[2] Biffoni M, Scipioni P, Macrina N, Amabile MI (2009). Pilonidal sinus. Outpatient treatment with local anesthesia. G. Chir.

[3] Ertan T, Koc M, Gocmen E, Aslar AK (2005). Does technique alter quality of life after pilonidal sinus surgery?. Am. J. Surg.

[4] Kepenekci I, Demirkan A, Celasin H, Gecim IE (2010). Unroofing and curettage for the treatment of acute and chronic pilonidal disease. World J. Surg.

[5] McCallum IJ, King PM, Bruce J (2008). Healing by primary closure versus open healing after surgery for pilonidal sinus: systematic review and meta-analysis. BMJ.

[6] Townsedn C, Evers M (2004). Sabiston-textbook of surgery.

[7] Spivak H (1996). Treatment of chronic pilonidal disease. Diseases of the Colon & Rectum.

[8] Akca T (2005). Randomized clinical trial comparing primary closure with the Limberg flap in the treatment of primary sacrococcygeal pilonidal disease. British Journal of Surgery.

[9] Thomas D (2002). Comparison of three methods in surgical treatment of pilonidal disease. ANZ Journal of Surgery.

[10] Seleem M, Al-Hashemy A (2005). Management of pilonidal sinus using fibrin glue: a new concept and preliminary experience. Colorectal Disease.

[11] Al-Khayat H (2007). Risk factors for wound complication in pilonidal sinus procedures. Journal of the American College of Surgeons.

[12] Lynch JB, Laing AJ, Regan PJ (2004). Vacuum-assisted closure therapy: a new treatment option for recurrent pilonidal sinus disease. Report of three cases. Diseases of the Colon & Rectum.

[13] Werkgartner G (2004). Knowledge-based therapy of the pilonidal sinus. European Surgery.

[14] Perruchoud C, Vuilleumier G (2002). Pilonidal sinus: how to choose between excision and open granulation versus excision and primary closure? Study of a series of 141 patients operated on from 1991 to 1995. Swiss surgery.

[15] McCallum I, King PM, Bruce J (2007). Healing by primary versus secondary intention after surgical treatment for pilonidal sinus. Cochrane Database Syst Rev.

[16] Ghafouri F (2011). A comparison between pilonidal sinus operation utilizing “Primary closure” and “Open method”. Iranian Magazine for Surgery.

[17] Sane’I B (2013). A comparison between the results of primary recovery of sacral pilonidal sinus surgery using Tie-over and Closed Suction Drainage techniques. Isfahan College of Medicine Magazine.

[18] Chintapatla S, Safarani N, Kumar S, Haboubi N (2003). Sacrococcygeal pilonidal sinus: historical review, pathological insight and surgical options. Tech. Coloproctol.

[19] Hølmebakk T, Nesbakken A (2005). Surgery for pilonidal disease. Scandinavian Journal of Surgery.

[20] Toccaceli S, Persico Stella L, Diana M, Dandolo R (2008). Treatment of pilonidal sinus with primary closure. Chir. Ital.

